# The MOVEMENT Trial

**DOI:** 10.1161/JAHA.118.010152

**Published:** 2019-01-13

**Authors:** Manne Holm, Per Tornvall, Loghman Henareh, Ulf Jensen, Nanna Golster, Patrik Alström, Irene Santos‐Pardo, Nils Witt, Nikolai Fedchenko, Dimitrios Venetsanos, Olof Beck, Jan van der Linden

**Affiliations:** ^1^ Department of Molecular Medicine and Surgery Karolinska Institutet Stockholm Sweden; ^2^ Perioperative Medicine and Intensive Care, B31 Karolinska University Hospital Huddinge, Stockholm Sweden; ^3^ Unit of Cardiology Department of Clinical Science and Education Södersjukhuset Karolinska Institutet Stockholm Sweden; ^4^ Coronary Artery and Vascular Disease Heart and Vascular Theme Department of Medicine Karolinska Institute and Karolinska University Hospital Stockholm Sweden; ^5^ Department of Laboratory Medicine Karolinska Institutet Stockholm Sweden

**Keywords:** angioplasty and stenting, antiplatelet therapy, myocardial infarction, narcotic antagonists, opioid, pharmacodynamics, pharmacokinetics, purinergic P2Y receptor antagonists, ST‐segment–elevation myocardial infarction, Clinical Studies, Platelets, Myocardial Infarction, Percutaneous Coronary Intervention, Treatment

## Abstract

**Background:**

Morphine administration is a strong predictor of delayed onset of action of orally administered ticagrelor in patients with ST‐segment–elevation myocardial infarction, likely because of impaired gastrointestinal motility. The aim of this study was to evaluate whether the peripheral opioid antagonist methylnaltrexone could improve pharmacodynamics and pharmacokinetics of orally administered ticagrelor in patients with ST‐segment–elevation myocardial infarction receiving morphine.

**Methods and Results:**

The MOVEMENT (Methylnaltrexone to Improve Platelet Inhibition of Ticagrelor in Morphine‐Treated Patients With ST‐Segment Elevation Myocardial Infarction) trial was a multicenter, prospective, randomized, controlled trial in patients with ST‐segment–elevation myocardial infarction treated with morphine and ticagrelor. Upon arrival to the catheterization laboratory, patients were randomized to a blinded intravenous injection of either methylnaltrexone (8 or 12 mg according to weight) or 0.9% sodium chloride. The proportion of patients with high on‐treatment platelet reactivity and plasma concentrations of ticagrelor and AR‐C124910XX were assessed at baseline (arrival in the catheterization laboratory) and 1 and 2 hours later. A total of 82 patients received either methylnaltrexone (n=43) or placebo (n=39). Median (interquartile range) time from ticagrelor administration to randomization was 41 (31–50) versus 45.5 (37–60) minutes (*P*=0.16). Intravenous methylnaltrexone administration did not significantly affect prevalence of high on‐treatment platelet reactivity at 2 hours after inclusion, the primary end point, when compared with placebo (54% versus 51%, *P*=0.84). Plasma concentrations of ticagrelor and its active metabolite, the prespecified secondary end points, did not differ significantly between the groups over time. There was no significant difference in patient self‐estimated pain between the groups.

**Conclusions:**

Methylnaltrexone did not significantly improve platelet reactivity or plasma concentrations of orally administered ticagrelor in patients with ST‐segment–elevation myocardial infarction receiving morphine.

**Clinical Trial Registration:**

URL: http://www.clinicaltrials.gov. Unique identifier: NCT02942550.

**Clinical Perspective**

**What Is New?**

- Intravenous administration of the peripheral opioid antagonist methylnaltrexone is likely not a viable method to improve the pharmacodynamics and pharmacokinetics of orally administered ticagrelor in patients with ST‐segment–elevation myocardial infarction receiving morphine.

**What Are the Clinical Implications?**

- The optimal way to counteract the morphine‐induced delay in ticagrelor among patients with ST‐segment–elevation myocardial infarction needs to be evaluated using other strategies than with intravenous methylnaltrexone in further studies.

## Introduction

A loading dose of ticagrelor is currently recommended in addition to aspirin as antiplatelet therapy in patients presenting with ST‐segment–elevation myocardial infarction (STEMI).^1^, ^2^ Administration of a loading dose of ticagrelor results in fast and potent platelet inhibition in patients with stable angina and non‐STEMI.^3^, ^4^ However, in patients with STEMI, a delayed onset of action and a wide variability of drug responses have been demonstrated after orally administered ticagrelor.^5^, ^6^ High on‐treatment platelet reactivity (HPR) is a risk factor for poor clinical outcome in patients undergoing percutaneous coronary intervention (PCI).^7^ In the ATLANTIC (Administration of Ticagrelor in the Cath Lab or in the Ambulance for New ST Elevation Myocardial Infarction to Open the Coronary Artery) study, prehospital ticagrelor administration did not improve pre‐PCI coronary reperfusion, but stent thrombosis was significantly lower at 30 days, when compared with administration of in‐hospital ticagrelor.^8^ Moreover, use of the intravenous P2Y12 inhibitor cangrelor in patients with STEMI has shown that an early and strong antiplatelet effect improves clinical outcome.^9^ It is thus important to continue to improve the onset of action of ticagrelor in patients with STEMI, as its effect improves clinical outcome.

The rate of drug absorption in the gastrointestinal tract is to a large extent determined by the rate of gastric emptying. Morphine, which is frequently administered to relieve pain in patients with STEMI is known to delay gastric emptying and has emerged as a predictor of delayed/poor antiplatelet response in patients receiving oral ticagrelor.^5^ Moreover, observational data have shown negative effects of morphine on clinical outcomes in patients with acute coronary syndrome (ACS).^10^ In a substudy of the ATLANTIC study, nonrandomized use of morphine in patients with STEMI was shown to delay the onset of platelet inhibition after a loading dose of ticagrelor.^11^ The authors hypothesized that this interaction may have had an impact on the outcome of the ATLANTIC study, where there was an observed interaction between morphine treatment and ST‐segment elevation resolution.^8^ In another recent trial, patients with acute myocardial infarction (64% STEMI) randomized to administration of morphine presented with a delayed uptake and antiplatelet response to ticagrelor, when compared with placebo.^12^ A negative effect on ticagrelor uptake and effect has also been shown for fentanyl.^13^

The morphine‐induced delay in gastric emptying can be reduced with the opioid antagonist naloxone, which has been verified in morphine‐treated women during labor.^14^ However, the drawback with naloxone is that it crosses the blood‐brain barrier and thereby reduces the pain‐relieving effects of morphine. In contrast, the morphine antagonist methylnaltrexone does not affect the morphine‐mediated central analgesic effects because of a reduced passage over the blood‐brain barrier, and thus primarily acts as a peripheral morphine antagonist^15^ without significantly different effectiveness and safety in the treatment of opioid‐related constipation compared with naloxone.^16^

Our hypothesis was that methylnaltrexone may improve pharmacodynamics and pharmacokinetics of orally administered ticagrelor in patients receiving morphine. The aim was to evaluate this in morphine‐treated patients with STEMI undergoing PCI, where rapid and adequate platelet inhibition after administration of ticagrelor is crucial.

## Methods

The MOVEMENT (Methylnaltrexone to Improve Platelet Inhibition of Ticagrelor in Morphine‐Treated Patients With ST‐Segment–Elevation Myocardial Infarction) trial (NCT02942550) was a prospective, single‐blinded, randomized, placebo‐controlled, multicenter study performed at Södersjukhuset and Karolinska University Hospital Huddinge in Stockholm, Sweden. Patient inclusion was performed between November 2016 and December 2017. The trial was approved by the regional ethical review board in Stockholm (institutional review board reference number 2015/1911‐31/4), and the Swedish Medical Product Agency approved the study as it was a clinical trial (EU‐no. 2015‐002910‐65). The data, analytic methods, and study materials will be available to other researchers for purposes of reproducing the results or replicating the procedure by contacting the corresponding author.

### Study Group

Patients considered for inclusion in the study were P2Y12 inhibitor–naive patients with STEMI presenting at the cardiac catheterization laboratory. Inclusion criteria were: (1) diagnosis of STEMI including presence of typical symptoms, eg, chest pain for >15 minutes together with new ST‐segment elevation (>1 mm [0.1 mV]) in at least 2 contiguous leads in the absence of left branch bundle block or signs of left ventricular hypertrophy on 12‐lead ECG; (2) intake of a 180‐mg oral loading dose of integral ticagrelor tablets given before initiation of coronary angiography; and (3) analgesic treatment with intravenous morphine administered before initiation of coronary angiography.

Exclusion criteria were: (1) cardiac arrest; (2) body weight >114 kg; (3) vomiting after oral intake of ticagrelor loading dose; (4) treatment with naloxone before inclusion or during the sampling period; (5) inability to understand study outline and instructions; (6) any methylnaltrexone bromide contraindication, including known hypersensitivity to the active substance or to any of the excipients and/or known or suspected mechanical gastrointestinal obstruction or other acute surgical abdominal conditions; (7) age younger than 18 years; (8) women in fertile age; (9) administration of ticagrelor, clopidogrel, or prasugrel within 7 days before onset of STEMI symptoms; (10) treatment with cangrelor; and (11) ongoing long‐term opioid treatment.

### Study Procedures

After oral consent was provided upon arrival to the cardiac catheterization laboratory, patients who fulfilled all of the inclusion criteria and no exclusion criteria were randomized to either active treatment with methylnaltrexone or placebo, as specified below. All patients were asked to provide written informed consent after completion of PCI. Randomization was performed thorough presealed envelopes in a 1:1 fashion in blocks of 4 patients. To facilitate reproducibility, randomization was performed with the tool available at www.randomization.com, which enables simple block randomization using equal fixed block sizes. Stratification was performed for the 2 participating centers and for inferior versus anterior or lateral STEMI, as acute inferior myocardial infarction often induces vagal enhancement with transient sinus bradycardia (Bezold‐Jarisch reflex), which is explained by preferential distribution of vagal nerve in the inferior wall.^17^ This may further impair gastrointestinal motility and possibly delay the absorption of peroral drugs. Data on the impact of this will be presented separately.

The responsible personnel in the cardiac catheterization laboratory administered the study drug methylnaltrexone (Relistor) or placebo after initial blood sampling. Methylnaltrexone was given as a single intravenous injection of 8 mg (0.4 mL solution) to patients weighing 38 to 61 kg or 12 mg (0.6 mL solution) to patients weighing 62 to 114 kg. The methylnaltrexone injection was given together with 10 mL of saline to flush the venous catheter. The placebo treatment of 0.9% sodium chloride was given as a single intravenous injection of 0.4 or 0.6 mL according to the same weight schedule as the study drug. The study drug or placebo was administered using an unlabeled injection syringe, thus blinding patients to their respective treatment. Patients were asked to state their self‐estimated pain using a visual analog scale (VAS) at baseline and at 1 and 2 hours after study intervention. The patients were asked to specify their level of pain by indicating a position along a continuous line between no pain (0/10) up to maximal pain (10/10).

### Platelet Function Testing and Drug Concentrations Measurements

At arrival to the catheterization laboratory, an initial baseline blood sample was drawn from the arterial introducer to avoid possible sampling failure that could conceivably delay the PCI. Further blood sampling was performed 1 and 2 hours after the patients received either methylnaltrexone or placebo. Blood samples were placed in 0.109 mol/L trisodium citrate tubes for pharmacodynamic evaluation. Blood samples were stored at room temperature and activated within 48 hours and lysed by the participating research nurses according to the manufacturer's instructions. The blood samples were then immediately frozen and stored below −20°C until analysis, as previously described.^11^ Analysis of P2Y12 inhibition blinded for study drug/placebo was performed centrally at Södersjukhuset by determination of platelet reactivity index with an ELISA assay for the measurement of phosphorylated vasodilator‐associated stimulated phosphoprotein (VASP) (CY‐QUANT VASP/P2Y12, BioCytex).

For pharmacokinetic assessment, blood samples were collected into lithium‐heparin tubes and then cooled before centrifugation at 1500*g* for 10 minutes at 4°C. The resulting plasma samples were then stored at the Södersjukhuset biobank below −20°C until analyzed. Plasma concentrations of ticagrelor and its active metabolite, AR‐C124910XX, were determined by a liquid chromatography high‐resolution mass spectrometry method and followed an earlier published procedure.^18^ Details regarding the analysis are described in Data S1. The lower limits of detection were 5.0 ng/mL for both ticagrelor and AR‐C124910XX. Plasma concentrations of morphine were determined using the same method. Values below the lower limit of detection were treated as zero in the statistical analysis.

### End Points

The primary end point was the prevalence of HPR, defined as platelet reactivity index ≥50% determined by the VASP assay^19^ 2 hours after randomization and subsequent intravenous injection of either study drug or placebo. Secondary end points were: (1) differences in ticagrelor and AR‐C124910XX concentrations at 1 and 2 hours after randomization; (2) differences in platelet reactivity index at 1 and 2 hours after randomization; and (3) differences in patients’ subjective pain according to visual analog scale.

**Figure 1:**
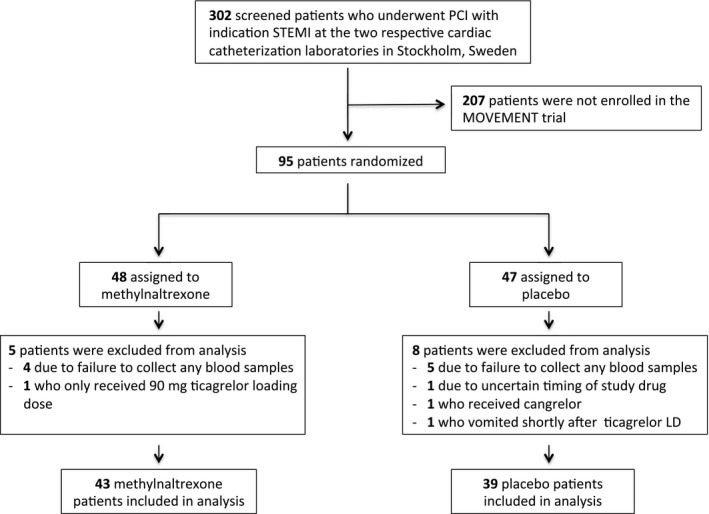
Enrollment and randomization. Patients who presented with ST‐segment–elevation myocardial infarction (STEMI) at the respective cardiac catheterization laboratories were considered for study inclusion. The figure shows the number of patients enrolled in the study and randomized. LD indicates Loading Dose; MOVEMENT, Methylnaltrexone to Improve Platelet Inhibition of Ticagrelor in Morphine‐Treated Patients With ST‐Segment–Elevation Myocardial Infarction; PCI, percutaneous coronary intervention.

Serious adverse events were registered within 48 hours after drug administration, corresponding to 5 half‐lives of methylnaltrexone, after which the remaining drug concentrations are considered negligible after a single dose.^20^ Vomiting and antiemetic treatment for nausea was also registered. Serious adverse events were reported for all patients who were randomized (n=95), ie, also for the patients who were excluded from the analysis (n=13) (Figure 1.

### Statistical Analysis

The sample size calculation was based on results from a previous substudy of the ATLANTIC study,^11^ which showed that patients with STEMI treated with morphine had a delayed onset of platelet inhibition after a 180‐mg loading dose of ticagrelor. In patients treated with morphine, 14 of 22 patients (64%) had HPR 3 hours after study inclusion (around 2 hours after PCI). Among the patients with STEMI who did not receive morphine, 3 of 15 patients (20%) had an HPR 3 hours after study inclusion. A sample size calculation showed that at least 25 patients would be needed in each group to obtain a significant difference between the groups (*P*<0.05, 90% power). To allow for stratification according to inferior or lateral/anterior STEMI, we aimed to include 40 patients in each group.

Categorical variables are presented as number (percentage) and compared using chi‐square test, or Fisher exact test if needed. Because of the relatively small sample size, and because many variables were nonnormally distributed, continuous variables are expressed as median and interquartile range with analysis between groups using the Wilcoxon rank sum test. In the respective tables, the frequency of missing data is reported. No imputation or other replacement of missing data was performed. A 2‐sided *P*<0.05 was considered significant. Statistical analyses were performed with SPSS version 24.0 (IBM) and Stata Statistical Software, release 14 (StataCorp).

## Results

### Patient Characteristics

During the study period, a total of 95 patients with STEMI were included in the study (12 patients at Karolinska University Hospital and 83 patients at Södersjukhuset), which represents 31% of the 302 patients who presented with STEMI at the 2 participating cardiac catheterization laboratories. The remaining 207 patients were not included mainly because of nonparticipating physicians on call. As shown in Figure 1, the final study cohort consisted of 82 patients who were randomly assigned to receive either methylnaltrexone (n=43) or placebo (n=39).

Baseline characteristics of the patients were well balanced between the groups, as shown in Table** 1. The median morphine dose did not significantly differ between the groups. Procedural characteristics are shown in Table** 2. The median time from the ticagrelor loading dose to study intervention was 45.5 (37–60) minutes for the methylnaltrexone group compared with 41 (31–50) minutes for the placebo group (*P*=0.16). The median additional morphine administered was not significantly higher in the methylnaltrexone group compared with the placebo group.

**Table 1:** Demographic Characteristics

Characteristic	MD	Placebo (n=39)	Methylnaltrexone (n=43)	*P* Value
Demographic/clinical				
Age, y	0	69 (58, 77)	64 (60, 73)	0.45
Age >75 y	0	12 (31)	7 (16)	0.12
Male sex	0	34 (87)	35 (81)	0.47
BMI	0	26.9 (25.0–29.1)	26.3 (24.1–28.1)	0.27
BMI >25	0	29 (74)	27 (63)	0.26
Hypertension	0	17 (44)	22 (51)	0.49
Diabetes mellitus	0	6 (15)	6 (14)	0.85
Dyslipidemia	0	8 (21)	9 (21)	0.96
Current smoker	0	5 (13)	13 (30)	0.06
Prior AMI	0	4 (10)	4 (9)	1.00
Prior PCI	0	4 (10)	4 (9)	1.00
Prior CABG	0	0 (0)	1 (2)	1.00
Prior nonhemorrhagic stroke	0	0 (0)	2 (5)	0.50
PAD	0	1 (3)	1 (2)	1.00
Chronic renal failure	0	2 (5)	2 (5)	1.00
COPD	0	2 (5)	4 (9)	0.68
Laboratory data				
Creatinine, μmol/L	0	83 (72–98)	82 (74–98)	0.92
eGFR, mL/min*	0	72 (59–84)	73 (58–82)	0.97
Hemoglobin g/L	0	147 (134–154)	147 (134–155)	0.88
Platelet count, ×10^9^	0	248 (201–288)	247 (208–285)	0.94
Prehospital medication				
Morphine dose, mg	2	6 (5–10)	6 (5–10)	0.63
Ondansetron	0	8 (21)	3 (7)	0.07
Metoclopramide	0	3 (8)	4 (9)	0.79

Continuous variables are described as median (interquartile range) and were tested with the Wilcoxon rank sum test. Categorical variables are described as number (percentage) and were tested with chi‐square test or with Fisher exact test if needed. AMI indicates acute myocardial infarction; BMI, body mass index; CABG, coronary artery bypass graft; COPD, chronic obstructive pulmonary disease; MD, missing data; PAD, peripheral arterial disease; PCI, percutaneous coronary intervention.

* Estimated glomerular filtration (eGFR) was calculated using the Cockcroft‐Gault formula.

**Table 2:** Procedural Characteristics

Procedural Characteristics	MD	Placebo (n=39)	Methylnaltrexone (n=43)	*P* Value
Clinical presentation				
Inferior STEMI	0	18 (46)	20 (47)	0.97
Systolic blood pressure, mm Hg	0	140 (125–155)	135 (120–152)	0.58
Cardiogenic shock	0	0 (0)	1 (2)	1.00
Pulmonary edema	0	2 (6)	0 (0)	0.24
Procedural aspects				
Time from ticagrelor LD to study intervention, min	1	41 (31–50)	45.5 (37–60)	0.16
Glycoprotein IIb/IIIa inhibitors	0	1 (3)	2 (5)	1.00
Heparin dose, IU	0	5000 (3000–8000)	5000 (3000–8000)	0.75
Enoxaparin	0	1 (3)	0 (0)	0.48
Bivalirudin	0	16 (41)	18 (42)	0.94
Thrombus aspiration	0	3 (8)	6 (14)	0.49
No. of stents used	0	1 (1, 2)	1 (1, 2)	0.92
Additional procedural medication				
Ondansetron	0	3 (8)	4 (9)	1.00
Metoclopramide	0	8 (21)	8 (19)	1.00
Morphine dose, mg	0	0 (0–3)	2 (0–5)	0.25

Continuous variables are described as median (interquartile range) and were tested with the Wilcoxon rank sum test. Categorical variables are described as number (percentage) and were tested with chi‐square test or with Fisher exact test if needed. LD indicates Loading Dose; MD, missing data; STEMI, ST‐segment–elevation myocardial infarction.

### Pharmacodynamic Response

**Figure 2:**
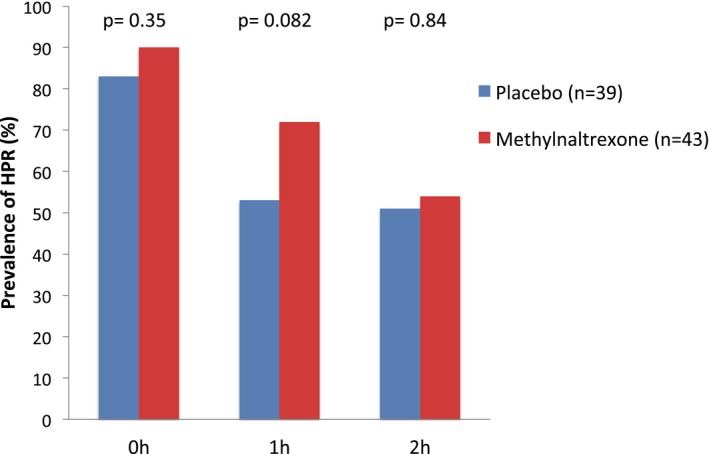
Prevalence of high on‐treatment platelet reactivity (HPR). Defined as ≥50% platelet reactivity index with the vasodilator‐associated stimulated phosphoprotein assay before inclusion and 1  and 2 hours after study intervention. Differences in the prevalence in HPR were tested with chi‐square test.

As shown in Table 3 and Figure 2, the primary outcome variable prevalence of HPR at 2 hours did not differ significantly between patients randomized to methylnaltrexone compared with placebo (54% versus 51%). We did have some missing data as a result of missed blood samples, as seen in Table 3. Additional pharmacodynamic data are presented in Table 3. Assessment with VASP showed no differences in platelet reactivity index percentage between methylnaltrexone and placebo at baseline (*P*=0.29) and at 1 (*P*=0.066) or at 2 hours (*P*=0.38) after the study intervention.

**Table 3:** Pharmacodynamic Evaluation

	MD	Placebo (n=39)	Methylnaltrexone (n=43)	*P* Value
Platelet function testing				
PRI% at baseline (IQR)	4	86.4 (67.0–92.8)	90.3 (68.7–93.8)	0.29
PRI% at 1 h (IQR)	8	59.0 (27.9–89.3)	84.9 (43.0–92.6)	0.066
PRI% at 2 h (IQR)	8	57.8 (20.9–84.1)	63.2 (24.3–89.2)	0.38
ΔPRI% 0 to 1 h (IQR)	5	14.9 (−1.40 to 39.0)	3.32 (−0.72 to 23.0)	0.18
ΔPRI% 0 to 2 h (IQR)	9	16.4 (−0.04 to 45.1)	11.6 (0.82–37.9)	0.73
HPR at baseline*	4	30 (83% [CI, 67–94%])	38 (90% [CI, 77–97%])	0.35
HPR at 1 h	8	18 (53% [CI, 35–70%])	29 (72% [CI, 56–85%])	0.082
HPR at 2 h	8	18 (51% [CI, 34–68%])	21 (54% [CI, 37–70%])	0.84

Continuous variables are described as median (interquartile range [IQR]) and were tested with the Wilcoxon rank sum test. Categorical variables are described as number (percentage) with 95% CIs and were tested with chi‐square test or with Fisher exact test if needed. The difference (Δ) in platelet reactivity index (PRI) percentage (PRI%) between the time points 0 to 1 hours and 0 to 2 hours is presented. Positive values suggest decreased PRI% levels. MD indicates missing data.

* High on‐treatment platelet reactivity (HPR) is defined as a PRI ≥50%.

### Pharmacokinetic Response

The drug concentration analyses of morphine, ticagrelor, and the main active metabolite of ticagrelor, AR‐C124910XX, are shown in Table 4 including comparisons between the groups. Plasma morphine concentrations did not differ significantly between the groups. Administration of methylnaltrexone when compared with placebo did not result in a significant difference in ticagrelor or AR‐C124910XX concentrations at any of the time points.

**Table 4:** Pharmacokinetic Evaluation

	MD	Placebo (n=39)	Methylnaltrexone (n=43)	*P* Value
Drug concentration analyses				
Morphine at baseline, ng/mL	0	12.8 (9.37–22.0)	13.2 (6.27–28.5)	0.94
Ticagrelor at baseline, ng/mL	0	0 (0–33.1)	0 (0–36.6)	0.42
Ticagrelor at 1 h, ng/mL	4	41.1 (0–571)	39.2 (0–154)	0.41
Ticagrelor at 2 h, ng/mL	5	88.3 (15.2–820)	105 (0–518)	0.88
ΔTicagrelor 1 to 0 h, ng/mL	4	22.7 (0–211)	27.5 (0–101)	0.81
ΔTicagrelor 2 to 0 h, ng/mL	5	40.2 (0–432)	94.6 (0–289)	0.57
AR‐C124910XX at 0 h, ng/mL	0	0 (0–0)	0 (0–0)	0.94
AR‐C124910XX at 1 h, ng/mL	4	0 (0–52.9)	0 (0–6.81)	0.17
AR‐C124910XX at 2 h, ng/mL	5	5.49 (0–104)	6.14 (0–55.9)	0.95
ΔAR‐C124910XX 1 to 0 h, ng/mL	4	0 (0–46.7)	0 (0–6.81)	0.15
ΔAR‐C124910XX 2 to 0 h, ng/mL	5	2.63 (0–94.8)	6.14 (0–54.1)	0.71

Continuous variables are described as median (interquartile range) and were tested with the Wilcoxon rank sum test. Categorical variables are described as number (percentage) and were tested with chi‐square test or with Fisher exact test if needed. The lower level of detection for ticagrelor and AR‐C124910XX concentrations were 5 ng/mL. The difference (Δ) in ticagrelor and AR‐C124910XX concentrations, respectively, between the time points 0 to 1 h and 0 to 2 h is presented. Positive values suggest increased concentrations. MD indicates missing data.

### Pain Perception

Morphine was the only analgesic drug used in the prehospital setting and during the first 2 hours of blood sampling when pain was also evaluated. Self‐estimated pain was unfortunately not registered and thus missing for 1 patient at baseline, 4 at 1 hour, and 8 at 2 hours after study inclusion. There was no difference in patient self‐estimated pain between patients receiving methylnaltrexone and placebo at baseline (median pain level [interquartile range] 3 [2–5] versus 2 [1–5], *P*=0.092]) or at 1 (0 [0–2] versus 1 [0–2], *P*=0.36) or 2 (0 [0–1] versus 0 [0–1], *P*=0.28) hours after the study intervention. Moreover, the difference in pain between baseline and 1 or 2 hours did not differ significantly between the groups (2 [0–4] versus 1 [0–3], *P*=0.085) and (2 [0–5] versus 2 [0–4], *P*=0.25, respectively).

### Adverse Events Within 48 Hours

Life‐threatening arrhythmia, defined as causing need for cardiopulmonary resuscitation, or nonsustained ventricular tachycardia did not differ significantly between the methylnaltrexone and placebo groups (6/48 [13%] versus 1/47 [2%], *P*=0.11; 29/48 [60%] versus 22/47 [49%], *P*=0.18). One patient in the methylnaltrexone group died from cardiac arrest during transportation to another hospital 2 hours after PCI. None of the patients in the placebo group died within 48 hours of PCI. The incidences of periprocedural/postprocedural pulmonary edema within 48 hours were 0/48 (0%) and 2/47 (4%; *P*=0.24), respectively. No patients experienced stent thrombosis, stroke, or bleedings other than from the access site. Nausea in need of antiemetic treatment with either metoclopramide or ondansetron was similar between patients with methylnaltrexone and placebo (10/48 [21%] versus 10/47 [21%], *P*=0.96; and 4/48 [8%] versus 4/47 [9%], *P*=0.98, respectively). Two patients in the methylnaltrexone group died after 48 hours during the hospital stay, 1 that was caused by sepsis and 1 that was caused by tracheal tube occlusion. The 3 deaths in the methylnaltrexone group during the hospital stay were reported to the responsible agencies, although neither the responsible physician nor the authors believe this was related to the use of methylnaltrexone. No other serious adverse event occurred within the 48‐hour period.

## Discussion

This randomized placebo‐controlled clinical trial involving patients with STEMI receiving intravenous morphine and a loading dose of 180 mg ticagrelor showed no significant effect of intravenous methylnaltrexone administration on the prevalence of HPR at 2 hours after inclusion when compared with placebo. These findings were supported by the pharmacokinetic measurements of ticagrelor and AR‐C124910XX concentrations. Pain, according to visual analog scale, did not differ significantly at baseline or at 1 and 2 hours between the 2 groups.

To our knowledge, the use of a peripheral opioid antagonist such as methylnaltrexone has not been studied among morphine‐treated patients with STEMI and there are thus no direct comparable previous study results. However, preliminary results have recently been presented as an abstract from a study in patients with angiographically documented coronary artery disease who were randomized to either intravenous methylnaltrexone or placebo before receiving intravenous morphine and a subsequent loading dose of 180 mg ticagrelor using a crossover design.^21^ In line with our results, the pharmacodynamic and pharmacokinetic analyses of that study showed no significant differences between methylnaltrexone and placebo at 30 minutes and 1, 2, 4, or 6 hours after the ticagrelor loading dose.^21^ Thus, the use of methylnaltrexone did not significantly improve ticagrelor uptake and effect in a nonacute setting or in the acute setting of STEMI.

Pain relief in ACS is of great importance in order to reduce the pain‐induced sympathetic activation resulting in elevated heart rate and blood pressure. This positive effect of morphine has, however, not been systematically tested in patients with ACS.^22^ On the contrary, observational data suggest negative effects of morphine administration in ACS with regards to clinical outcome.^8^, ^10^, ^23^ This could at least in part be explained by the negative impact of morphine on the uptake and effect of oral P2Y12 inhibitors, as shown in several studies and mentioned in the 2017 European Society of Cardiology STEMI guidelines.^24^ In the present study, a majority of patients had HPR at 2 hours after the study intervention, when assessed with the VASP assay. This is in line with previous studies on morphine‐treated patients with ACS, where assessment of platelet function was performed using the VASP assay.^11^, ^12^ Early and strong antiplatelet effect is important, as shown with the intravenous P2Y12 inhibitor cangrelor.^9^ In the ATLANTIC study, stent thrombosis was also significantly lower at 30 days in the group with prehospital ticagrelor, when compared with in‐hospital ticagrelor.^8^ Thus, there is a need for improvement of early platelet inhibition in patients with STEMI to minimize the risk for thrombotic events.

In the present study, adverse events within 48 hours did not differ significantly between the groups. There was indeed a numerically higher incidence of life‐threatening arrhythmia in the patients receiving methylnaltrexone. This may lack statistical significance as a result of a type II error. However, in an assessment report published by the European Medicines Agency, the observed incidence of serious adverse events in patients taking long‐term subcutaneous methylnaltrexone did not raise any safety concerns.^25^ Nevertheless, one should remain vigilant regarding cardiovascular events in possible future studies.

Possible strategies to overcome impaired early platelet inhibition in patients undergoing PCI include crushing or chewing of ticagrelor tablets,^26^, ^27^ use of an intravenous P2Y12 inhibitor such as cangrelor,^9^ or perhaps addition of an antiemetic agent such as metoclopramide (NCT02627950). The use of other opioids, such as fentanyl, has also been shown to impair ticagrelor uptake and onset of effect.^13^ The optimal management in this respect, however, needs to be evaluated in further studies. Additional studies on the peripheral opioid antagonist methylnaltrexone should be avoided as administration of this substance has so far failed to show any sign of improving ticagrelor uptake in morphine‐treated patients with STEMI.

### Study Limitations

A number of limitations of the MOVEMENT trial should be noted. The sample size was not sufficient to address any impact on clinical outcome. However, sample size calculation was performed based on available data, and our study was powered to provide significant differences regarding our primary end point. It may, however, be noted that the power calculation used was related to ticagrelor uptake in morphine versus no morphine, while the study evaluated ticagrelor uptake in morphine versus morphine and methylnaltrexone, as no data on the latter were available at the start of the study. Another limitation is that we did not exclude patients taking antiemetic treatment, which has prokinetic properties on gastric emptying.^28^ Moreover, we did not have reliable data on the time from symptom onset to study inclusion.

The dose recommendation for subcutaneous methylnaltrexone is 8 or 12 mg according to the summary of the product characteristics available from the European Medicines Agency. As poor subcutaneous perfusion was deemed likely in patients with STEMI, an intravenous administration was chosen to avoid inadequate methylnaltrexone blood concentrations, which was approved by the Swedish Medical Products Agency. Clinical experience and published data indicate that 8 or 12 mg of subcutaneous methylnaltrexone result in bowel movement for constipated patients with high opioid doses (>100 mg/d).^29^ As the summary of the product characteristics states that the absolute bioavailability of a subcutaneous dose is 82% compared with the same intravenous dose, no dose adjustment was performed.

However, the summary of the product characteristics refers to publications where 0.30 mg/kg methylnaltrexone was given intravenously. In our study, the intravenous dose of 8 or 12 mg methylnaltrexone resulted in a mean dose of 0.15 mg/kg. It is conceivable, but unlikely, that a higher dose may have improved the ticagrelor uptake and antiplatelet effect, especially as no tendency was shown toward any beneficial effect on ticagrelor uptake and effect in our study. Moreover, the higher dose of 0.30 mg/kg methylnaltrexone did not result in any beneficial effect on the ticagrelor uptake and antiplatelet effect as compared with placebo in patients with angiographically documented coronary artery disease.^21^

Patients who vomited were excluded from the study as they always received a second ticagrelor loading dose, making interpretation of drug concentrations and platelet inhibition difficult. This may be considered a limitation. Another limitation is the in‐hospital study inclusion and methylnaltrexone/placebo injection. Since the Swedish Medical Products Agency requires a physician asking for study participation, this approach was chosen. A prehospital administration of methylnaltrexone, perhaps concurrent with morphine, may have resulted in improved uptake of ticagrelor. However, this is unlikely, as in the abstract discussed above, methylnaltrexone administration before morphine and ticagrelor administration did not significantly improve ticagrelor uptake or platelet inhibition compared with placebo.^21^ Blood sampling was conducted until 2 hours after the study intervention, which corresponds to almost 3 hours after oral intake of ticagrelor. Previous studies have shown the ticagrelor maximal concentrations of healthy volunteers and patients with non‐STEMI not receiving opioids were at 2 hours after oral intake.^3^, ^4^ In contrast, most patients with STEMI have adequate effect of ticagrelor 4 to 6 hours after the loading dose.^5^, ^6^ Our hypothesis was that methylnaltrexone would reverse the negative impact of morphine with a significant improvement in early platelet inhibition, which is why early blood sampling was deemed of greatest value. Moreover, this improved the feasibility of the study. Thus, there is a possibility that a later difference in onset of ticagrelor effect might have occurred, which is a limitation. This, however, seems unlikely considering the previous reports presenting results up to 6 hours after the ticagrelor loading dose without a significant difference between methylnaltrexone and placebo.^21^ Moreover, in the IMPRESSION (Influence of Morphine on Pharmacokinetics and Pharmacodynamics of Ticagrelor in Patients With Acute Myocardial Infarction) trial, a significant difference in the prevalence of HPR between morphine and placebo was seen already at 30 minutes for the VASP assay.^12^ Only 1 platelet function test was used, which might also be considered a limitation, although VASP is a well‐established method. The pharmacodynamic results are, however, in line with the ticagrelor and ARC124910XX concentrations.

### Study Strengths

An important strength of the present study is the analysis of morphine concentrations at baseline before the study intervention and that there was no significant difference between the groups. The time from the ticagrelor loading dose and the baseline ticagrelor concentrations did not differ significantly between the groups, which is important as this is another possible source of bias. In addition, the trial was randomized and placebo‐controlled, with relatively few exclusion criteria.

## Conclusions

Methylnaltrexone did not significantly improve platelet reactivity or plasma concentrations of orally administered ticagrelor in patients with STEMI receiving morphine.

## Sources of Funding

Funding was provided by a grant from the Swedish Heart and Lung Foundation.

## Disclosures

None.

## Supporting information


**Data S1.** Supplemental methods.
