# Supplementary material for: The MOVEMENT Trial
Source: J Am Heart Assoc. 2019 Jan 13;8(2):e010152. doi: 10.1161/JAHA.118.010152 (PMC6497337; doi:10.1161/JAHA.118.010152)

SUPPLEMENTAL MATERIAL

Data S1.

Supplemental Methods

Drug concentration measurements

Plasma concentrations of ticagrelor, its metabolite and morphine were determined with a liquid chromatography – high resolution mass spectrometry (LC-HRMS) method.

The used instrument was a UHPLC-Q Exactive high resolution mass spectrometer (Thermo Scientific, Waltham, MA, USA) equipped with a Dionex 3000 UltiMate LC system consisting of an ultra-high pressure dual pump, an auto-sampler, solvent degasser and a thermostated column oven. The TraceFinder software v4.2 was used for instrument control and data evaluation.

After thawing, 0.1 mL of plasma was added into a 7 mL glass test-tube. Thereafter, 0.2 mL of acetonitrile containing internal standards (10 ng ticagrelor-d7, 10 ng ticagrelor metabolite-d7, 8 ng morphine-d3) was added during vortexing. The prepared sample was centrifuged at 3400 x g for 5min and 150 µL was transferred into a new glass-test tube. After evaporation to dryness in a vacuum centrifuge the residue was redissolved in 60 µL of 50% acetonitrile (10% for morphine) and transferred into an autosampler vial.

A volume of 1 µL was injected into the LC-HRMS system. Chromatographic separation was achieved using an Hypersil C18 column (particle size 1.9 µm, 2.1 mm x 100 mm, Thermo Scientific) operating at a column temperature of 40°C, and using gradient elution with a buffer A (0.1 % formic acid in water) and a buffer B (0.1% formic acid in acetonitrile) with a flow rate of 0.5 mL/min. The MS was operated in full scan positive electrospray ionization mode at 70,000 resolution setting. Extracted ion

chromatograms with 10 ppm tolerance were used for peak area measurements. The exact masses of the protonated molecules were m/z 523.1934 for ticagrelor and 530.2373 for the d7 analogue, m/z 479.1671 for ticagrelor metabolite and 486.2111 for the d7 analogue, m/z 286.1438 for morphine and 289.1626 for the d3 analogue.

Calibration curves using fortified plasma standards were using the following concentrations: 5, 30, 100, 200, 600, 1200, 2000 ng/mL for ticagrelor and ticagrelor dealkylated metabolite (ALSAChim, Strasbourg, France), and 2, 12, 40, 80 ng/mL for morphine (Cerriliant Co, Round Rock, Texas, USA). Internal standrards were from the same companies. The correlation coefficients (*r*) of the respective calibration curves generated during the validation was >0.999 for all three analytes.

The quality controls run together with the study samples had the following results:

Analyte	Assigned concentration, ng/mL	Measured concentration, ng/mL	CV(%)	N
Ticagrelor	15	17.0	5.5	10
	180	195	5.1	10
	1000	1131	4.6	10
Metabolite	15	14.0	6.4	10
	180	188	1.9	10
	1000	1069	1.6	10
Morphine	6	5.6	2.5	10
	72	69	2.2	10

Representative chromatograms (analyte and internal standard):

Ticagrelor metabolite 40 ng/mL

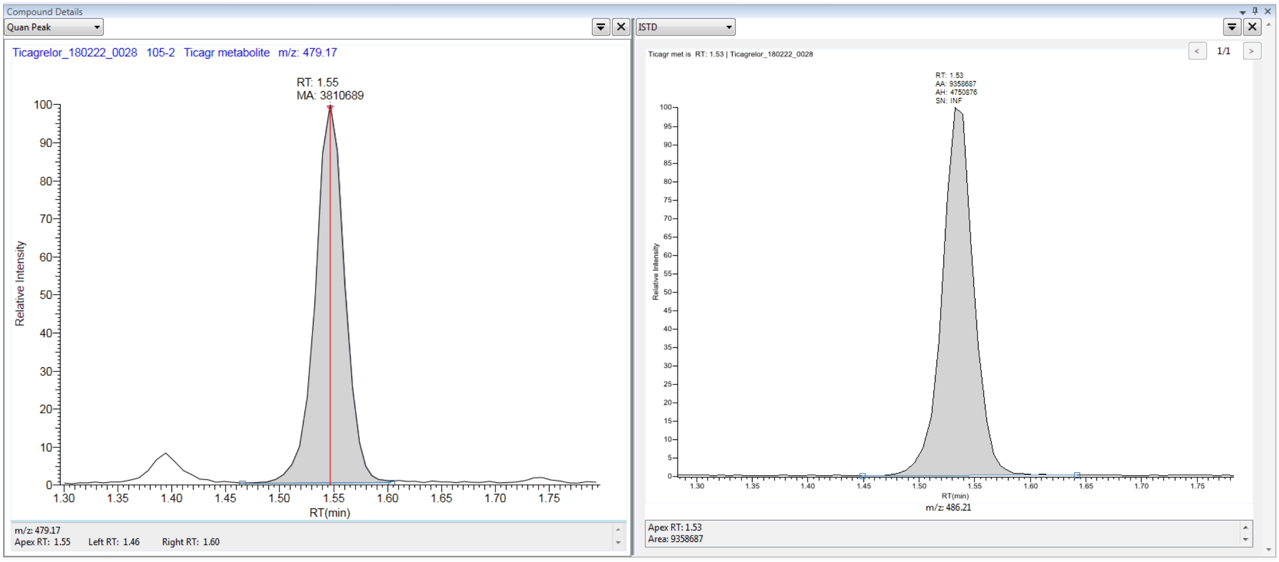

Ticagrelor 518 ng/mL

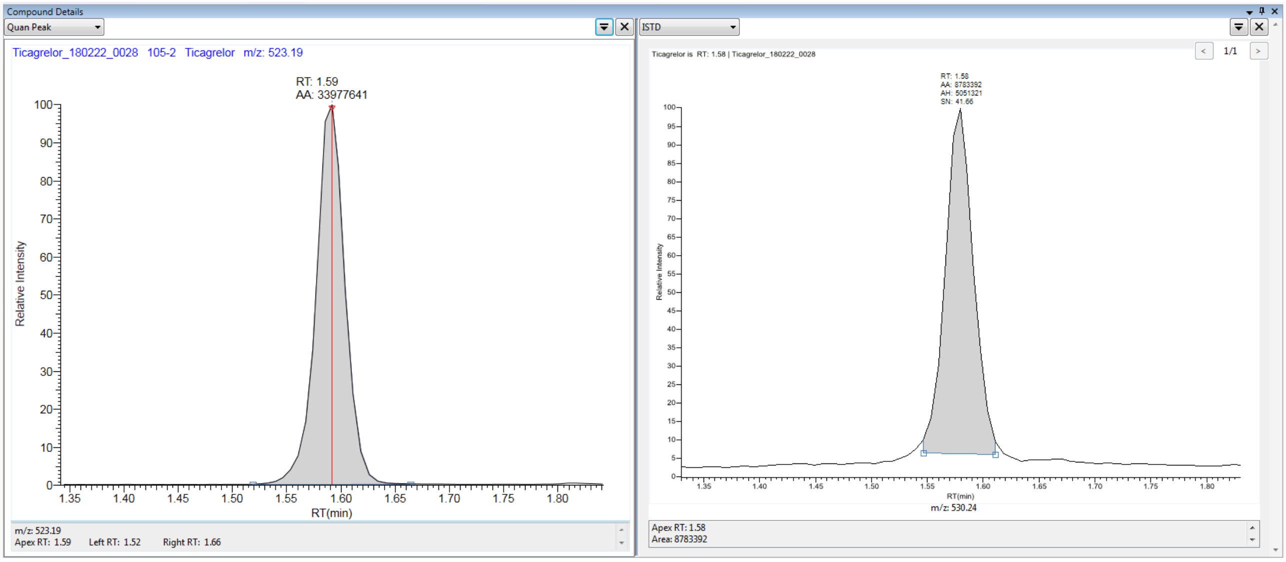

Supplement: Supplementary file 1 — Data S1. Supplemental methods. [file JAH3-8-e010152-s001.pdf]
